# Brazilian task force for the management of mucormycosis

**DOI:** 10.1016/j.bjid.2025.104579

**Published:** 2025-09-19

**Authors:** Patrick Leon de Godoy Macedo, Mariane Taborda, Vítor Falcão de Oliveira, Adriana Satie Gonçalves Kono Magri, Lígia Lins Frutuoso, Gideane Mendes de Oliveira, Sinaida T. Martins, Daniel Wagner de Castro Lima Santos, Fabianne Altruda de Moraes Costa Carlesse, Francelise Bridi Cavassin, Kelsen Dantas Eulálio, Marcia Lazera Andréa, Andréa d’Avila Freitas, José Ernesto Vidal, Dayvison Francis Saraiva Freitas, Marcia Garnica, Terezinha do Menino Jesus Silva Leitão, Rosely Maria Zancopé-Oliveira, Marcia de Souza Carvalho Melhem, Flavio Queiroz Telles, Maria Aparecida Shikanai-Yasuda, Fernanda Dockhorn Costa, Maria Adelaide Millington, Marcello Mihailenko Chaves Magri

**Affiliations:** a Hospital das Clínicas da Faculdade de Medicina da Universidade de São Paulo (HCFMUSP), Departamento de Infectologia e Medicina Tropical, São Paulo, SP, Brazil; b Ministério da Saúde, Coordenação Geral de Vigilância da Tuberculose, Micoses Endêmicas e Micobactérias não Tuberculosas (CGTM/DATHI/SVSA/MS), Brasília, DF, Brazil; c Hospital Universitário da Universidade Federal do Maranhão (HU-UFMA/EBSERH), Departamento de Moléstias Infecciosas, São Luís, MA, Brazil; d Rede D'Or, Hospital UDI, Intituto D'Or de Pesquisa e Ensino (IDOR), São Luís, MA, Brazil; e Universidade Federal de São Paulo, Escola Paulista de Medicina (UNIFESP/EPM), São Paulo, SP, Brazil; f Universidade Federal de São Paulo, Instituto de Oncologia Pediátrica (IOP) GRAACC/UNIFESP, São Paulo, SP, Brazil; g Faculdades Pequeno Príncipe, Curitiba, PR, Brazil; h Universidade Federal do Piauí, Departamento de Doenças Infecciosas e Parasitárias, Terezina, PI, Brazil; i Fundação Oswaldo Cruz, Instituto Nacional de Infectologia Evandro Chagas, Laboratório de Micologia, Rio de Janeiro, RJ, Brazil; j Instituto de Infectologia Emílio Ribas, Departamento de Neurologia, São Paulo, SP, Brazil; k Fundação Oswaldo Cruz, Instituto Nacional de Infectologia Evandro Chagas, Laboratório de Pesquisa Clínica em Dermatologia Infecciosa, Rio de Janeiro, RJ, Brazil; l Universidade Federal do Rio de Janeiro, Rio de Janeiro, RJ, Brazil; m Universidade Federal do Ceará, Hospital São José de Doenças Infecciosas, Departamento de Saúde Comunitária, Fortaleza, CE, Brazil; n Universidade Federal do Mato Grosso do Sul, Faculdade de Medicina, Campo Grande, MS, Brazil; o Universidade de São Paulo, Faculdade de Medicina, Laboratório de Investigação Médica LIM53, São Paulo, SP, Brazil

**Keywords:** Mucormycosis, Liposomal amphotericin B, Surgical debridement, Isavuconazole, Ministry of health, Task force

## Abstract

**Background:**

Mucormycosis is a rare but life‑threatening fungal infection that has shown an increased incidence in Brazil, especially during the COVID‑19 pandemic.

**Objective:**

To provide an evidence‑based, context‑specific guideline for the diagnosis and management of mucormycosis within the Brazilian healthcare system. Clinical features: Rhino‑orbito‑cerebral disease predominates, followed by pulmonary, cutaneous, gastrointestinal and disseminated forms; delayed recognition dramatically increases mortality.

**Epidemiology:**

The global incidence of mucormycosis is increasing, particularly among patients with diabetes mellitus, hematologic malignancies, transplantation, and corticosteroid exposure. The most frequently isolated species is Rhizopus arrhizus, and regional variations in species distribution may be present. In Brazil, comprehensive epidemiological data remain scarce.

**Treatment:**

Early, aggressive surgical debridement plus induction with liposomal amphotericin B (5–10 mg/kg/day) followed by isavuconazole or posaconazole is recommended; strict control of hyperglycemia and immunosuppression is essential.

**Conclusion:**

Standardized national guidance, improved rapid diagnostics, systematic surveillance and equitable drug availability are critical to reduce Brazil’s mucormycosis burden.

## Introduction

Mucormycosis is a rare but highly invasive fungal infection caused by fungi of the order Mucorales, associated with significant morbidity and mortality rates ranging from 25 % to 80 %. It primarily affects diabetic and immunocompromised individuals and manifests in various forms including Rhino-Orbito-Cerebral (ROC), pulmonary, cutaneous, gastrointestinal, and disseminated conditions. Among these, the ROC form is the most prevalent presentation, particularly in patients with uncontrolled diabetes mellitus.^1^, ^2^, ^3^ In Brazil, 311 cases of mucormycosis were reported between 2010 and 2021, with a notable surge during the COVID-19 pandemic, which saw 85 cases predominantly in São Paulo, affecting individuals over 40 years old and primarily presenting as ROC.^4^

The increased incidence during the pandemic can be attributed to factors such as indiscriminate corticosteroid use and glycemic dysregulation.^5^, ^6^, ^7^, ^8^, ^9^ Early diagnosis is critical for reducing mortality, with diagnostic modalities including imaging studies, histopathological examination, and microbiological cultures.^3^^,^^10^, ^11^, ^12^, ^13^, ^14^ The cornerstone of treatment involves a combination of aggressive surgical debridement and antifungal therapy, with the recommended regimen comprising Liposomal Amphotericin B (L-AmB) as induction therapy, followed by Isavuconazole (ISA) for sequential therapy.^3^ The Brazilian Ministry of Health facilitates access to l-AmB and ISA for the treatment of mucormycosis through the public health system.

Given the complexities of diagnosis and management, a comprehensive approach is essential. This includes addressing underlying predisposing factors such as hyperglycemia and immunosuppression to improve clinical outcomes.^3^ In light of these challenges, the formation of a task force to develop evidence-based guidelines for the diagnosis and management of mucormycosis in Brazil is crucial. This narrative review aims to synthesize current literature, identify gaps in knowledge, provide actionable recommendations to enhance the clinical care of affected patients and improve outcomes and reduce the burden of this severe opportunistic infection across the country.

Although the recommendations are tailored to the realities and constraints of the Brazilian Unified Health System (SUS), they may also serve as a reference for other middle-income countries. In higher-resource settings, adaptations may be required, particularly regarding access to advanced diagnostics and newer antifungal agents.

## Materials and methods

This narrative review was developed by a task force composed of specialists in mycology, infectious diseases, and epidemiology, members of The Advisory Technical Committee on Endemic and Opportunistic Mycoses (CTA-MIC) of the Ministry of Health, with expertise in the management of invasive fungal infections, including mucormycosis. For the development of this article, the group was expanded to include infectious disease specialists from the Medical Mycology Group of the Hospital das Clínicas, Faculty of Medicine, University of São Paulo (HC-FMUSP), and a pediatric infectious disease specialist from the Federal University of São Paulo (UNIFESP). Conflicts of interest were declared and recorded.

Clinical questions were formulated using the PICO model (Population, Intervention, Comparator, and Outcome),^14^ addressing critical aspects of mucormycosis management, such as therapeutic options, timing of intervention, and the impact of adjunctive measures (Table 1). These questions were reviewed by the expert group and prioritized based on clinical relevance and variability in practice. The responses were formulated in consensus meetings, where HC-FMUSP specialists discussed the evidence profiles considering benefits, risks, and clinical applicability. The draft recommendations were then submitted to the expert panel from the Ministry of Health and the pediatric infectious diseases specialist for review and suggestions.

**Table 1:** Levels of Evidence and Strength of Recommendations according to the GRADE System.

Clinical Question (PICO)	Quality of Evidence	Strength of Recommendation	References
In patients with mucormycosis, does the use of high-dose liposomal amphotericin B (L-AmB) as induction therapy improve survival compared to standard-dose l-AmB?	Moderate (Experimental studies, RCTs unavailable, but moderate observational evidence)	Strong (Highly recommended in several international guidelines); High dose l-AmB is recommended as first-line induction therapy.	^65^^,^^67^^,^^69^ Experimental studies^77^^,-^^80^:
Should combination therapy be considered for patients with mucormycosis, as compared to treatment with amphotericin B (AmB) alone?	Low (Limited to case reports, a few observational studies, a recent systematic review, but conflicting results).	Moderate (A recent systematic review found that the combination of AmB and azoles was associated with significantly lower mortality); Combination therapy with AmB and azoles may be an alternative.	^34^^,^^66^^,^^81^, ^82^, ^83^, ^84^, ^85^ Experimental studies^86^^,-^^87^:
In patients with mucormycosis, does sequential therapy with triazoles improve clinical outcomes compared to standard management?	Moderate (Limited data from clinical studies, some observational support, and a recent systematic review)	Trong (A recent systematic review found that sequential therapy of AmB followed by azole had lower mortality); Isavuconazole is recommended for sequential therapy.	^6^^,^^34^^,^^85^^,^^88^^,^^89^
^In patients with mucormycosis undergoing surgical debridement, does early intervention improve survival compared to delayed surgery?^	^High (Multiple observational studies with consistent findings)^	^Strong (Early surgical debridement is associated with better outcomes)^	^7^^,^^10^^,^^21^^,^^25^^,^^40^^,^^92^^,^^100^, ^101^, ^102^, ^103^, ^104^, ^105^, ^106^, ^107^, ^108^, ^109^, ^110^, ^111^, ^112^

GRADE (Grading of Recommendations, Assessment, Development, and Evaluation) system.^20^^,^^21^

A narrative literature review was conducted in MEDLINE/PubMed, Embase, and LILACS without restrictions on period or date, in addition to selected articles from Cornely et al. (2019).^3^ Two reviewers (M.M.C.M. and P.L.G.M.) independently screened titles/abstracts and full texts. When disagreement occurred, a third reviewer (M.T.) adjudicated by consensus.

Studies were included if they met the following criteria: population (patients with confirmed or suspected mucormycosis); intervention (antifungal agents, surgery, and adjunctive strategies); comparator (standard treatment or different therapeutic approaches, especially combination therapy). Outcomes: mortality, clinical response, safety, and adverse events. The certainty of the evidence was graded according to the GRADE (Grading of Recommendations, Assessment, Development, and Evaluation) system^15^^,^^16^ as high, moderate, low, or very low (Table 1). A structured search was conducted in MEDLINE/PubMed, Embase, and LILACS to inform GRADE assessments addressing predefined PICO questions on treatment strategies for mucormycosis. Search terms included combinations of “mucormycosis” OR “zygomycosis” with keywords related to antifungal agents (e.g., “liposomal amphotericin B”, “isavuconazole”, “posaconazole”), treatment strategies (e.g., “combination therapy”, “surgical debridement”, “glycemic control”), and clinical outcomes. No language or date restrictions were applied, and filters were used to select human studies only. The recommendations consider the clinical practice context in Brazil, including resource availability, hospital infrastructure, and access to antifungal agents and diagnostic tests. The Key Points for the Management of Mucormycosis in Brazil are summarized in Table 2.

**Table 2:** Key points for the management of mucormycosis in Brazil.

Box 1: What are the Mucorales?
Mucormycosis is a rare but life-threatening fungal infection caused by thermotolerant fungi from the Mucorales order, with *Rhizopus* spp. being the predominant global causative agent.
Clinical forms vary by anatomical site and pathogen, with *Rhizopus* spp. linked to ROC disease.


Box 2: Who is at higher risk of developing mucormycosis?
Mucormycosis predominantly affects individuals with DM, HM, and transplant recipients, with geographic variations in risk factors.
Breakthrough mucormycosis occurs in up to 20 % of cases during antifungal therapy, with voriconazole more frequently associated with Mucorales-related infections.
Additional risk factors include iron overload, corticosteroid use, malnutrition, and HIV/AIDS, highlighting the multifactorial nature of the disease.


Box 3: Should we consider mucormycosis in Brazil?
The global incidence of mucormycosis has been rising, particularly in India and China, even before the COVID-19 pandemic.
CAM surged in India, with a disease burden higher than the global average, mainly affecting diabetic patients and linked to corticosteroid use.
In Brazil, mucormycosis cases increased during the pandemic, with most infections reported in São Paulo and predominantly in older adults with the ROC form.


Box 4: Pathogenesis of mucormycosis
Mucormycosis is primarily acquired through inhalation, with fungal adhesion and invasion mediated by CotH-GRP78 interactions and iron dysregulation.
Neutropenic and hyperglycemic states impair phagocytic function, facilitating fungal proliferation, while Mucorales exploit iron acquisition mechanisms for survival.
SARS-CoV-2 infection increases mucormycosis susceptibility via hyperglycemia, iron overload, metabolic acidosis, and GRP78 overexpression in epithelial cells.


Box 5: What are the main signs and symptoms of the primary clinical forms?
ROCM can rapidly progress from sinus infection to orbital and cerebral involvement, leading to thrombosis, infarcts, and high mortality.
Pulmonary mucormycosis, often seen in hematologic malignancies and transplant recipients, presents with nodules, cavitations, and the characteristic reverse halo sign.
Cutaneous, gastrointestinal, and disseminated forms vary in presentation but share high lethality, particularly in immunocompromised patients.


Box 6: What are the main differential diagnoses for mucormycosis? Why is it important to recognize them?
Mucormycosis differentials vary by clinical form, requiring thorough evaluation with imaging, histopathology, and microbiology.
ROCM must be distinguished from bacterial sinusitis, *Aspergillus* spp., *Fusarium* spp., granulomatous diseases, and neoplasms.
Pulmonary and gastrointestinal forms overlap with fungal infections, mycobacteriosis, endemic mycoses, and malignancies, making early recognition crucial for timely antifungal therapy.


Box 7: Under what circumstances should ROC mucormycosis be suspected? What are the definitions of probable and proven mucormycosis according to the Revision and Update of the Consensus Definitions of Invasive Fungal Disease by the EORTC and the MSG and Research Consortium?
ROCM should be suspected in high-risk patients, especially those with DM, HM, neutropenia, or recent COVID-19, presenting with facial pain, necrotic ulcers, or ocular symptoms.
Probable mucormycosis requires compatible clinical features, a predisposing condition, and mycological evidence from a non-sterile
site.
Proven mucormycosis is confirmed by histopathology or a positive culture/PCR from a sterile site, demonstrating tissue invasion.


Box 8: How to diagnose and treat mucormycosis in Brazil and middle-income countries?
Mucormycosis is a medical emergency requiring rapid diagnosis via imaging (CT/MRI), endoscopy, histopathology, and culture, with molecular testing showing promise in BAL and serum.
Radiology is essential for staging disease progression, with MRI preferred for ROC involvement and chest CT recommended for pulmonary cases, highlighting the reverse halo sign.
Histopathology confirms diagnosis by identifying broad, non-septate hyphae with right-angle branching, while culture is crucial for species identification and treatment guidance.


Box 9: How to treat mucormycosis in Brazil and middle-income countries?
Mucormycosis treatment relies on three pillars: early antifungal therapy, aggressive surgical debridement, and prompt control of underlying risk factors.
Liposomal amphotericin B (5‒10 mg/kg/day) remains the cornerstone of induction therapy, with isavuconazole or posaconazole recommended for sequential therapy.
Effective management requires collaboration among infectious disease specialists, surgeons, radiologists, pathologists, and intensive
care teams.
Treatment protocols should consider local epidemiological data and recommendations. Brazilian initiatives and task forces aim to standardize and improve the management of mucormycosis.
Continuous monitoring for therapeutic efficacy, potential side effects of antifungal agents, and complications is essential, along with supportive care tailored to the patient's clinical status.
Continued research into novel diagnostic and therapeutic strategies, along with training for healthcare professionals, is vital to advancing care for mucormycosis in Brazil.

ROCM, Rhino-Orbito-Cerebral Mucormycosis.

## Answers to the questions

### What are the mucorales?

Mucormycosis is a rare, opportunistic fungal infection characterized by invasive growth caused by filamentous, hyaline fungi with coenocytic hyphae of the order Mucorales. Historically referred to as Zygomycosis, however, following advancements in fungal phylogenetics, this classification was discontinued.^17^ Mucorales are thermotolerant fungi with a ubiquitous distribution, commonly found in natural environments, including fruits, decaying organic matter, starch-rich foods, molds and soil.^18^^,^^19^

In humans, approximately 11 genera and 27 species are associated with mucormycosis.^1^ The distribution of these genera and species is influenced by geographic location, climatic variations, underlying host conditions, and routes of infection.^2^ The genera most commonly implicated in mucormycosis include *Rhizopus, Mucor,* and *Lichtheimia*. Among these, *Rhizopus* spp. contribute to the majority of cases globally with *R. arrhizus* being the most prevalent species.^20^, ^21^, ^22^, ^23^, ^24^, ^25^ *Lichtheimia* spp. have been identified as the predominant causative agents in certain hospitals in Spain and other parts of Europe.^21^^,^^23^^,^^25^^,^^26^ *Cunninghamella, Apophysomyces, Saksenaea, Rhizomucor, Cokeromyces, Actinomucor*, and *Syncephalastrum* have also been implicated in mucormycosis cases worldwide.^1^^,^^2^ Notably, *Apophysomyces* species are a significant secondary cause of mucormycosis in India.^19^^,^^20^

Clinical forms are classified based on the affected anatomical site into ROC, pulmonary, Gastrointestinal (GIM), cutaneous, renal, disseminated, and other diverse forms, including infections in bones, heart, ear, parotid gland, uterus, urinary bladder, and lymph nodes.^1^, ^2^, ^3^

A meta-analysis revealed associations between mucormycosis agents and its clinical forms. *Rhizopus* spp. are frequently associated with the ROC form, while *Cunninghamella* spp. are more commonly linked to pulmonary or disseminated disease, and *Apophysomyces* and *Saksenaea* species are often isolated in cutaneous forms.^10^^,^^22^ Diabetic ketoacidosis predisposes individuals to infections by *Rhizopus* spp. but less so by *Lichtheimia*.^10^^,^^21^^,^^23^^,^^25^^,^^26^ Mortality associated with *Cunninghamella* spp. is significantly higher compared to other genera.^10^^,^^22^

### Who is at higher risk of developing mucormycosis?

Mucormycosis is associated with vascular invasion, thrombosis, and dissemination, leading to high morbidity and mortality rates, averaging 25 % and ranging from 40 % to 80 %.^3^^,^^27^ The infection is most common among patients with DM, neutropenia, HM, Solid Organ Transplants (SOT), Hematopoietic Stem Cell Transplants (HSCT), and corticosteroid therapy.^10^^,^^20^, ^21^, ^22^, ^23^, ^24^, ^25^, ^26^, ^27^, ^28^, ^29^, ^30^^,^^31^, ^32^, ^33^, ^34^ In recent years, mucormycosis has also been associated with healthcare settings.^28^ DM is the most common risk factor in Asia^22^ and Latin America,^24^ whereas HM and transplants are predominant in Europe and the United States.^10^^,^^20^, ^21^, ^22^, ^23^, ^24^, ^25^, ^26^, ^27^

Mucormycosis occurs as a breakthrough infection in patients with HM or those undergoing HSCT who are receiving antifungal prophylaxis with triazoles or echinocandins.^35^^,^^36^ Some studies suggest an association between the use of Voriconazole (VCZ) for prophylaxis or treatment and an increased incidence of mucormycosis, while others do not support this finding.^37^, ^38^, ^39^ A recent comprehensive systematic review and meta-analysis on breakthrough Invasive Fungal Infections (bIFIs), screened 5293 studies for eligibility, ultimately selecting 300 studies for detailed data extraction.^36^ These studies documented 1076 cases of bIFIs that developed during antifungal therapy with either VCZ (42.5 %) or Posaconazole (PCZ) (57.5 %). The predominant pathogens identified were *Aspergillus* (40 %), Mucorales (20 %), *Candida* (18 %), and *Fusarium* (9 %) species. A notable pathogen-specific pattern emerged: Mucorales were more commonly associated with VCZ-related bIFIs, whereas *Aspergillus* and *Fusarium* species were more frequently identified in cases occurring under PCZ prophylaxis.^36^

Other risk factors include HIV/AIDS infection, intravenous drug use, low-birth-weight neonates, malnutrition, chronic alcoholism, liver disease, chemotherapy, use of calcineurin inhibitors, iron overload and deferoxamine therapy.^40^, ^41^, ^42^

### Should we consider mucormycosis in Brazil?

The incidence of mucormycosis has been increasing globally, even before the COVID-19 pandemic, particularly in India and China, countries with high DM prevalence.^7^^,^^10^^,^^20^, ^21^, ^22^, ^23^, ^24^, ^25^^,^^43^ A recent review of 851 cases from January 2000 to January 2017 found that 34 % of reported cases were from Europe, followed by Asia (31 %), the Americas (28 %), Africa (3 %), and Australia/New Zealand (3 %).^22^ The true incidence/prevalence of mucormycosis may be even higher, as many cases remain undiagnosed due to difficulties in obtaining deep tissue samples, low sensitivity of diagnostic tests, and underreporting or non-reporting of cases in various regions.

During the COVID-19 pandemic, a significant increase in mucormycosis cases was reported in several countries, particularly in India, resulting in thousands of cases unprecedented in the history of this mycosis. The high incidence of COVID-19-Associated Mucormycosis (CAM) may be related to the high prevalence of DM in the Indian population, endemic fungal presence, climate, healthcare-related factors and inappropriate corticosteroid use for moderate to severe viral infection treatment.^5^, ^6^, ^7^, ^8^, ^9^^,^^44^^,^^45^ The most common clinical form was ROC, and the mortality rate was high at 49 %, particularly among patients with pulmonary or disseminated forms or cerebral involvement. A significant proportion of survivors had severe sequelae, such as vision loss, affecting 46 % of the survivors.^9^

In Brazil, mucormycosis is not classified as a mandatory notifiable disease. Since 2008, the Ministry of Health has distributed antifungal medications for the treatment of mucormycosis. Surveillance data come primarily from requests for antifungal therapy for mucormycosis. Between 2018 and June 14, 2021, these medications were provided for a total of 143 cases. By October 4, 2021, the Ministry recorded 90 cases of mucormycosis, with 47 of these cases linked to COVID-19. This number exceeds the cases reported in the previous years: 27 in 2018, 31 in 2019, and 35 in 2020 (https://www.gov.br/saude/pt-br/assuntos/saude-de-a-a-z/m/mucormicose/situacao-epidemiologica). A time-series study conducted from 2010 to 2021 identified a total of 311 cases, with 85 occurring during the pandemic. The majority of these cases were found in individuals over 40 years old (84 %), predominantly white (78 %), with the ROC form of the disease (63 %), and primarily residing in São Paulo State (84 %). The rise in reported cases may be associated with the increase in severe COVID-19 cases in Brazil, a trend also observed in other regions around the world.^4^

### Pathogenesis of mucormycosis

Humans primarily acquire the infection by inhaling environmental sporangiospores. Occasionally, transmission occurs through ingestion of contaminated food or implantation, particularly in immunocompetent individuals such as those with burns, traumatic wounds, or during calamities like earthquakes, hurricanes, and tornadoes.^1^^,^^18^

Fungal dissemination is facilitated by various virulence mechanisms inherent to these pathogens. Host factors, including key comorbidities and risk factors previously described, play a significant role in the disease's pathogenesis. The interaction between fungal spores and host endothelial cells is crucial for adhesion and invasion. This process involves the binding of the spore-coating protein (CotH) from the pathogen to Glucose-Regulated Protein 78 (GRP78) present on host cells.^2^

Conditions such as acidemia and hyperglycemia destabilize plasma iron chelators (ferritin and transferrin), leading to an excess of this ion. These changes promote increased GRP78 expression on endothelial surfaces, subsequently heightening the risk of fungal invasion. The first barrier in the human body is innate immunity. Neutrophils and macrophages trigger a pro-inflammatory response via Toll-Like Receptor 2 (TLR-2), leading to phagocytosis, oxidative metabolite production, and defensins. Neutropenic patients or those with dysfunctional phagocytes fail to control fungal proliferation. Hyperglycemia and acidemia, primarily found in decompensated diabetic patients, are critical factors causing phagocytic dysfunction. Fungal survival within the host primarily depends on its ability to acquire iron. The first mechanism involves the increased expression of genes encoding high-affinity iron-binding proteins (ferroxidase, ferropermease, and ferrireductase). The second mechanism relies on the production of siderophores (e.g., rhizoferrin) or the uptake of external siderophores, such as deferoxamine, used in patients undergoing dialysis for chronic renal disease. The third mechanism involves heme oxygenase activity, which captures iron from heme groups.^2^^,^^46^, ^47^, ^48^

COVID-19 infection can predispose individuals to mucormycosis through additional factors beyond those previously mentioned. Hyperglycemia may result from direct SARS-CoV-2 damage to pancreatic islets and insulin resistance caused by the cytokine storm and cortisol stress response. The direct action of the virus on the kidneys, combined with thrombosis and ischemia, can lead to acute kidney injury and metabolic acidosis. Excess ferritin synthesis, driven by elevated IL-6 secretion, increases iron availability. SARS-CoV-2 also induces GRP78 overexpression in nasal epithelial cells and facilitates the entry of fungal hyphae.^5^^,^^8^^,^^44^

### What are the main signs and symptoms of the primary clinical forms?

The ROC form often progresses beyond rhinosinusitis, invading the orbit and, subsequently, the brain parenchyma through thrombosis and ischemia. Common symptoms include fever, headache, facial edema, facial pain, facial numbness, nasal or palatal ulcers, bone destruction, nasal discharge, epistaxis, dental pain, facial nerve paralysis, hemiplegia, and altered consciousness levels. Key ophthalmologic signs and symptoms include ocular pain, vision loss, ophthalmoplegia, proptosis, ptosis, orbital cellulitis, periorbital discoloration, and necrosis.^1^, ^2^, ^3^ Imaging studies, such as CT and MRI, are essential for assessing tissue invasion extent.^3^ Typical findings in the sinuses include mucosal thickening, bone erosion, sinusitis, and bone destruction of the nasal septa, orbit, maxilla, and mandible. When orbital and cerebral extensions occur, manifestations may include orbital cellulitis, optic neuritis, soft tissue infiltration, rarefaction and erosion of the skull base, cavernous sinus thrombosis, internal carotid artery thrombosis, and intracranial infarcts or abscesses.^1^^,^^3^^,^^49^

A robust Mexican study (1982–2017) involving 250 patients proposed a management flowchart and highlighted warning signs, including cranial nerve paralysis, proptosis, periorbital edema, diplopia, sinus pain, ophthalmoplegia, and palatal ulceration.^50^

The pulmonary form is the second most common and is frequently observed in patients with HM, HSCT, transplant recipients, and DM.^1^^,^^3^^,^^20^^,^^22^ Symptoms include fever, cough, pleuritic chest pain, dyspnea, and hemoptysis. Imaging findings may be nonspecific, including multiple nodules, pulmonary consolidation, pleural effusion, thick-walled cavities, hilar or mediastinal lymphad enopathy, air crescent signs, pneumothorax, and the reverse halo sign characteristic of mucormycosis.^1^^,^^3^^,^^50^^,^^51^ Pulmonary mucormycosis is usually unilateral, with the upper lobe most commonly affected, followed by the lower and middle lobes.^51^

Cutaneous mucormycosis typically occurs following trauma or skin breaches and may be observed in immunocompetent hosts. The primary predisposing factor is penetrating trauma. Other risk factors include intramuscular injection, motor vehicle accidents, surgery, contaminated dressings, burns, natural disasters, animal bites, and scratches. Diabetic patients and transplant recipients may occasionally develop cutaneous mucormycosis.^52^, ^53^, ^54^

GIM is one of the most challenging forms to diagnose, predominantly seen in low-birth-weight neonates, malnourished individuals, or those on peritoneal dialysis. In classical immunocompromised hosts, the disease is more common in SOT patients, HM, and neutropenic individuals. Peritoneal dialysis and DM are significant factors in adults, while broad-spectrum antibiotics and malnutrition are significant factors in children. The intestine is the most common site, including the large intestine, stomach, small intestine, and esophagus. Symptoms include abdominal pain, bleeding, abdominal distension, and diarrhea.^55^^,^^56^ Cases of GIM have been infrequently reported in Brazil.^57^

Disseminated mucormycosis occurs hematogenously, most commonly in HM patients and transplant recipients. The lungs are the most frequent site of dissemination in over 90 % of cases, followed by the central nervous system, sinuses, liver, and kidneys.^1^^,^^3^^,^^10^^,^^21^

### What are the main differential diagnoses for mucormycosis? Why is it important to recognize them?

The differential diagnosis of mucormycosis depends on the underlying diseases and clinical presentations. There are several types of fungal rhinosinusitis, and the differential diagnosis may vary depending on clinical presentation and test results. Accurate differential diagnosis requires a comprehensive clinical evaluation, including medical history, physical examination, imaging results, biopsy of affected sinus tissues, direct mycology examination, and culture. Key considerations for differentiating fungal rhinosinusitis include:

A) For the ROC form:

1. Bacterial rhinosinusitis.
2. Other fungal rhinosinusitis caused by Hyalo-hyphomycoses (notably aspergillosis and fusariosis).
3. Other inflammatory or infectious nasal conditions such as nasal polyposis, vasculitis, granulomatous diseases like granulomatosis with polyangiitis, sarcoidosis, and various neoplasms like nasal sinus carcinoma and allergic rhinosinusitis.

B) For pulmonary forms, especially in diabetic and immunosuppressed patients with HM, transplants, neutropenia, and/or corticosteroid or other immunosuppressive drug:

1. Other Hyalo-hyphomycoses, particularly aspergillosis and fusariosis.
2. Mycobacteriosis and endemic diseases such as paracoccidioidomycosis, particularly with a reverse halo sign.
3. Obliterative bronchiolitis, bacterial pneumonia, pulmonary infarction, and lung neoplasms.

C) For gastrointestinal forms, mucormycosis is often a diagnosis of exclusion. Primary differentials include other causes of diarrhea, intestinal opportunistic infections, inflammatory bowel disease, mycobacteriosis, neoplasms, and acute abdomen (inflammatory, vascular, and obstructive causes).

Under what circumstances should ROC mucormycosis be suspected? What are the definitions of probable and proven mucormycosis according to the Revision and Update of the Consensus Definitions of Invasive Fungal Disease by the European Organization for Research and Treatment of Cancer (EORTC) and the Mycoses Study Group Education and Research Consortium?^11^

1. Suspected *mucormycosis:* should be considered in patients presenting with the following risk factors and clinical signs: a) *Risk Factors:* uncontrolled diabetes (particularly with ketoacidosis); HM; HSCT; prolonged neutropenia; SOT; corticosteroid use; recent COVID-19 infection; iron overload. b) *Signs and Symptoms:* Early Signs: acute, localized pain (including radiation to the eye); fever; general malaise; intense facial pain.^58^ Late Signs: nasal ulcers with black exudate; bleeding; facial edema and asymmetry; ocular pain, ptosis, visual disturbances, or blindness; bruising and necrosis around the nose; paranasal sinus extension into bony barriers, including the orbit and palate; neurological symptoms suggestive of central nervous system involvement.^3^^,^^11^^,^^49^

2. Probable mucormycosis: requires the presence of a host factor (e.g., immunosuppression or diabetes), clinical features consistent with mucormycosis, and mycological evidence (e.g., direct microscopy, culture, or PCR) from a non-sterile site.^11^

3. *Proven mucormycosis:* requires the demonstration of Mucorales hyphae in tissue by histopathology or cytology, showing evidence of tissue invasion, or a positive culture or PCR from a sterile site, along with clinical symptoms consistent with mucormycosis.^11^

### How to diagnose and treat mucormycosis in Brazil and other middle-income countries?

**Fig. 1:**
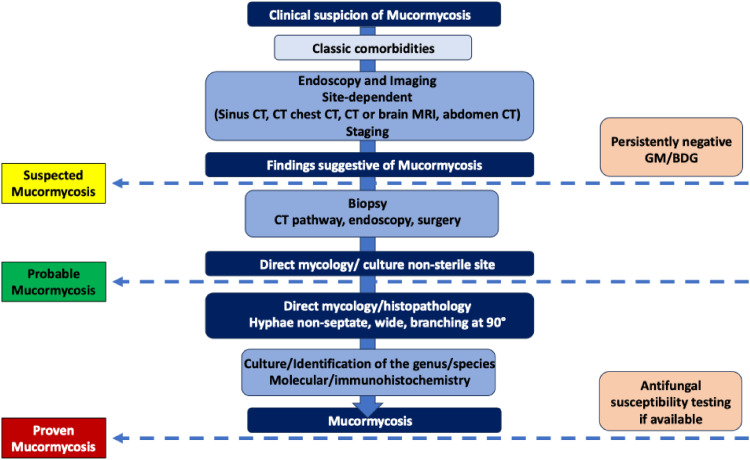
Diagnostic pathway and classification criteria for mucormycosis.

Suspected or confirmed mucormycosis is a medical emergency that demands rapid intervention to prevent angioinvasion, tissue necrosis, and dissemination. Timely intervention can reduce the extent of surgical debridement required and significantly enhance patient survival.^3^^,^^49^ Effective management depends on access to advanced imaging modalities (CT and/or MRI), endoscopic evaluations (e.g., nasofibroscopy, upper gastrointestinal endoscopy, or colonoscopy), a well-trained multidisciplinary team, and robust mycological and histopathological diagnostic capabilities.^3^ Fig. 1 summarizes the recommended diagnostic pathway for mucormycosis, from clinical suspicion to confirmation, integrating imaging, histopathology, and microbiological methods.

***a) What is the role of radiology in mucormycosis?***

For patients with symptoms suggestive of ROC mucormycosis, cranial CT or MRI is strongly recommended to detect and stage sinusitis.^3^^,^^12^ The most common radiographic finding is rhinosinusitis, which is often indistinguishable from bacterial infections. While mucosal thickening and partial or complete sinus opacification are frequently observed, the presence of bone erosion indicates disease progression. The absence of sinus involvement on CT has a high negative predictive value for ROC mucormycosis. The main stages of ROC progression include nasal mucosa involvement, extension to the perinasal sinuses, orbital invasion, and ultimately, Central Nervous System (CNS) involvement. In confirmed sinusitis, endoscopic or nasofibroscopic evaluation is strongly recommended to confirm the diagnosis of mucormycosis. For suspected orbital or cerebral involvement, MRI is preferred over CT due to its superior sensitivity.^3^^,^^11^, ^12^, ^13^^,^^59^ In immunocompromised patients, cranial, thoracic, and abdominal imaging is essential to determine the full extent of the disease. Serial and weekly imaging is recommended, particularly for surgically treated or clinically unstable patients, as it helps to monitor therapeutic response.^3^^,^^11^

For suspected pulmonary mucormycosis, chest CT is strongly recommended to identify characteristic findings, including multiple pulmonary nodules (typically >10), pleural effusion, and the reverse halo sign, defined as ground-glass opacity surrounded by a ring of consolidation. Pulmonary angiography may provide additional evidence by demonstrating vascular occlusion. Diagnostic confirmation often requires invasive procedures, such as CT-guided needle biopsy or BAL obtained via bronchoscopy, which should be utilized based on clinical feasibility and resource availability.^3^^,^^49^^,^^50^^,^^60^

***b) What are the main histological characteristics?***

Histopathology plays a crucial role in diagnosing mucormycosis. Confirmation requires identifying characteristic hyphae in tissue sections stained with Hematoxylin and Eosin (H&E), Periodic Acid-Schiff (PAS), or Grocott-Gomori methenamine silver stains. Histologically, the hyphae are typically broad, ribbon-like, and irregular, measuring 6–16 μm in width and occasionally exceeding 25 μm. They are coenocytic (non-septate) or sparsely septate, with right-angle (90°) branching. This contrasts with other hyaline molds, which exhibit acute angle branching and frequent septation. Artificial septations may appear due to tissue processing, and identifying right-angle branching can be challenging because of interstitial pressures and alterations in tissue architecture. Therefore, hyphal width and irregularity are more reliable diagnostic features than septation or branching angles. Immunohistochemistry with commercially available monoclonal antibodies can assist in uncertain cases. Additionally, PCR techniques on fresh or paraffin-embedded tissue exhibit high specificity for detecting Mucorales but require further standardization to improve diagnostic accuracy.^3^^,^^58^^,^^61^

***c) Should we request direct mycological examination, culture, and susceptibility testing?***

Culture is strongly recommended for the presumptive identification of Mucorales at the genus and species level and provides material for molecular conclusive species identification. Patients with palatal, sinus, or skin lesions should undergo biopsy for microscopic analysis, culture, and histopathological examination. In culture, Mucorales form grayish colonies with abundant mycelial growth. The isolation of Mucorales from tracheal or sinus secretions may indicate colonization or contamination; therefore, confirmation with direct microscopy and biopsy findings is crucial for accurate diagnosis.^58^^,^^61^ While antifungal susceptibility testing is not universally endorsed in the mucormycosis latest international guidelines (2019), it can be clinically valuable in cases of therapeutic failure.^3^

***d) Emerging promising diagnostic methods***

DNA detection in serum and other body fluids shows significant promise but requires further standardization. Molecular methods for clinical specimens, particularly BAL, are feasible and supported by the availability of commercial kits.^62^^,^^63^ Serum DNA detection, although not widely accessible in many centers, could serve as a valuable diagnostic tool. However, its applicability and reliability need validation within the Brazilian healthcare setting.

In Brazil, the diagnosis of mucormycosis remains a major challenge due to structural and logistical limitations that affect both timeliness and accuracy. Access to histopathology, direct mycological examination, and fungal culture is often restricted to reference centers, resulting in delayed or missed diagnoses in community and regional hospitals. Moreover, advanced diagnostic tools such as PCR-based assays and MALDI-TOF MS, which may improve early detection, are not widely available across the public healthcare system. The absence of standardized molecular platforms and the limited integration of rapid diagnostics into clinical workflows contribute to a median diagnostic delay of 7 to 10 days, as observed in many public institutions. This delay is particularly critical in mucormycosis, where prompt diagnosis directly influences survival. Strengthening laboratory capacity, decentralizing diagnostic technologies, and incorporating point-of-care molecular methods could significantly improve early recognition and outcomes, particularly in high-risk populations served by the SUS.

### Incorporation of L-AmB and ISA for the treatment of mucormycosis in Brazil and the advisory technical committee on endemic and opportunistic mycoses (CTA-MIC), ministry of health, Brazil

The inclusion by the Ministry of Health in Brazil of ISA in the Public Health System, along with the expanded use of l-AmB, was successfully achieved following the approval by the National Committee for Health Technology Incorporation in the SUS (Conitec). ISA was incorporated for the treatment of mucormycosis during the consolidation phase, replacing lipid formulations of amphotericin B. This recommendation was formalized in Final Report n° 745/2022, published on July 28, 2022, and officially established by Ordinance SCTIE/MS n° 73, dated September 1, 2022, and published on September 2, 2022.

The expanded use of l-AmB for patients with Rhino-Orbito-Cerebral (ROC), the most prevalent form of mucormycosis, was recommended in Final Report n° 287/2022, published on April 28, 2022. This expansion was implemented through Ordinance SCTIE/MS n° 57, dated June 23, 2022, and published on June 24, 2022. Both decisions were preceded by public consultations, which incorporated contributions from specialists and civil society, ensuring alignment with the needs of the Public Health System in Brazil (SUS). These advancements underscore the Ministry of Health's commitment to promoting evidence-based, highly effective treatments for severe fungal diseases.

The Advisory Technical Committee on Endemic and Opportunistic Mycoses (CTA-MIC) was established by the Ministry of Health through Ordinance GM/MS n° 3098, dated January 18, 2024, and officially published on January 19, 2024.

The CTA-MIC acts as an advisory board to the Department of HIV/AIDS, Tuberculosis, Viral Hepatitis, and Sexually Transmitted Infections of the Secretariat of Health Surveillance and Environment on technical and scientific matters pertaining to endemic and opportunistic mycoses. The CTA-MIC is composed of representatives from a range of esteemed institutions, including members from the Ministry of Health, the Oswaldo Cruz Foundation, the National Council of Health Secretaries, the National Council of Municipal Health Secretariats, the Pan American Health Organization, as well as members from scientific societies and leading experts in the field of medical mycology.

### How to treat mucormycosis in Brazil and other middle-income countries?

Treatment recommendations for mucormycosis are primarily derived from retrospective studies, systematic reviews, small uncontrolled prospective studies, and case-control reports. To date, no randomized controlled trials have been conducted for mucormycosis (Table 1). Additional challenges in managing this disease include the diversity of genera and species involved, each exhibiting varying virulence and antifungal susceptibility, geographic differences, distinct clinical scenarios, underlying conditions, and the variability of surgical approaches, which are often individualized.

***a) What are the three fundamental pillars for the treatment of mucormycosis?***

**Fig. 2:**
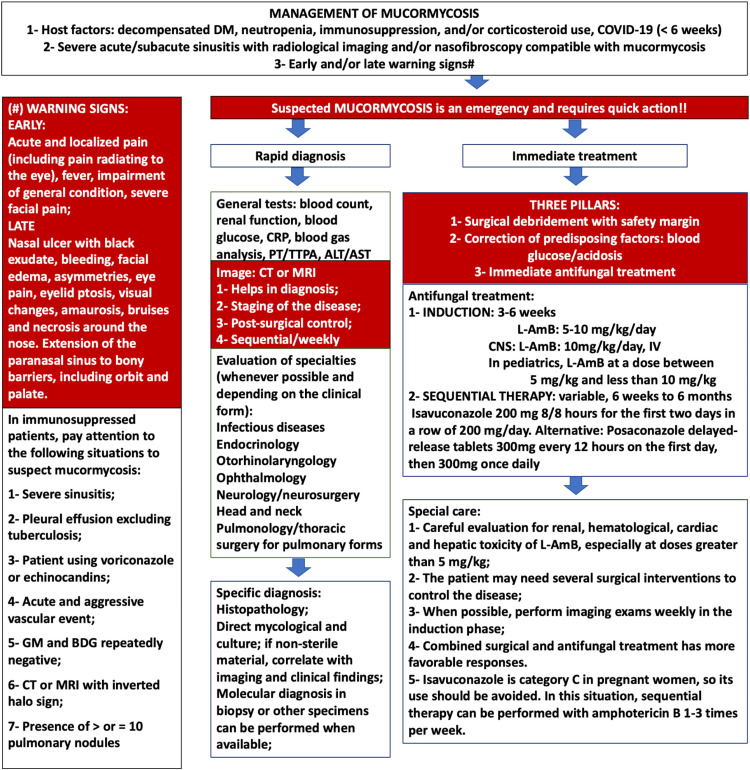
Steps for the management of mucormycosis in Brazil. ALT, Alanine Aminotransferase; AST, Aspartate Aminotranferase; BDG, Beta-d-Glucan; CNS, Central Nervous System; CRP, C-Reactive Protein; CT, Computed Tomography; GM, Galactomanan; l-AmB, Liposomal Amphotericin B; MRI, Magnetic Resonance Imaging; PT, Prothrombin Time; TTPA, Activated Partial Thromboplastin Time.

In November 2019, the European Confederation of Medical Mycology published updated diagnostic and treatment recommendations, which provide guidance on managing this complex and life-threatening infection. Optimal treatment of mucormycosis ideally involves a combination of aggressive surgical debridement, appropriate antifungal therapy, and reversal or control of predisposing factors, with a particular emphasis on glycemic control.^3^ The management strategies for mucormycosis are summarized in Fig. 2.

**1) Appropriate antifungal therapy** is defined as the prescription of the correct antifungal agent at the correct dose, initiated immediately, even upon suspicion of infection. Antifungal treatment consists of two phases: the induction phase and the sequential or consolidation phase. Accurate staging of the fungal infection is critical for therapeutic success, particularly when CNS involvement is suspected. The most commonly used antifungal agents in clinical practice include l-AmB, posaconazole, and ISA. l-AmB is considered highly effective for mucormycosis and requires higher-than-usual doses compared to those used for other invasive fungal infections, often administered over prolonged periods.^1^^,^^3^^,^^10^^,^^21^^,^^25^^,^^64^, ^65^, ^66^, ^67^, ^68^, ^69^, ^70^, ^71^, ^72^, ^73^, ^74^ ISA, a triazole antifungal agent, has been approved by the United States Food and Drug Administration (FDA) as a first-line treatment option for mucormycosis.^75^^,^^76^ In Brazil, the Ministry of Health provides l-AmB for the induction phase and ISA for the consolidation phase, ensuring access to optimal treatment regimens for this severe condition.

**a) Induction Phase** (3–6 weeks): l-AmB at 5–10 mg/kg/day, avoiding dose escalation. With CNS involvement: High doses of l-AmB at 10 mg/kg/day, supported by animal models^77^, ^78^, ^79^, ^80^ and human studies.^65^^,^^67^^,^^69^ Renal, cardiac, hepatic, and hematologic toxicity must be carefully monitored. ABLC 5 mg/kg per day is an option for patients without CNS involvement, while the Use of amphotericin B Deoxycholate (D-AmB) is not recommended. The 2019 global guideline recommends ISA as first-line therapy for patients with preexisting renal impairment. However, this should be approached cautiously, as most of the literature focuses on the use of AmB. Recommended doses for intravenous ISA: Loading dose of 200 mg IV every 8 h for 2 days, followed by 200 mg/day IV.^3^

### Should we perform combination therapy with l-AmB and triazoles (isavuconazole or posaconazole)?

The use of combination antifungal therapy, such as amphotericin B with echinocandins or triazoles, remains a topic of debate.^34^^,^^66^^,^^68^^,^^81^, ^82^, ^83^, ^84^, ^85^, ^86^, ^87^ Although retrospective analyses have not consistently demonstrated a significant benefit,^66^ a recent systematic review encompassing 126 articles published between 2000 and 2022 analyzed data from 5364 patients treated with antifungals. Statistical analysis revealed a significant advantage for combination therapy with AmB and triazoles, as well as for sequential therapy involving AmB followed by triazoles, compared to AmB monotherapy.^34^

Given the available evidence and the absence of randomized controlled trials, combination therapy could be considered in selected high-risk scenarios. This strategy may be appropriate for patients with renal dysfunction who cannot tolerate high doses of l-AmB, and as salvage therapy in cases of refractory or progressive disease despite appropriate monotherapy. In Brazil, combination regimens have been used by some specialists, particularly in hematology-oncology settings, in the context of severe or disseminated infections, therapeutic failure, or drug-related toxicity, especially nephrotoxicity.

**b) Sequential therapy:** The duration of therapy ranges from a minimum of 6-weeks to 3–6 months or longer, depending on clinical, surgical, and radiological criteria. The efficacy of triazoles in consolidation or salvage therapy is well-supported by international guidelines^3^^,^^34^^,^^71^, ^72^, ^73^, ^74^ and evidence from clinical studies.^6^^,^^34^^,^^85^^,^^88^^,^^89^ Recommended doses for oral ISA: loading dose of 200 mg three times daily (PO) for 2 days, followed by 200 mg once daily. Posaconazole delayed-release tablets are also an option for sequential therapy: loading dose of 300 mg every 12 h on the first day, then 300 mg once daily.

### How to treat mucormycosis in pediatric patients?

Recommendations for pediatric mucormycosis are almost entirely extrapolated from adult studies. Limited pediatric clinical trials exist, evidence comes mainly from case reports and case series, highlighting the need for dedicated research. The most frequently observed clinical forms include ROC, pulmonary, cutaneous, and GIM. Among neonates, GIM is the most commonly reported form, associated with particularly high mortality rates.^3^^,^^55^^,^^56^^,^^90^, ^91^, ^92^, ^93^, ^94^, ^95^ Therapeutic principles remain consistent across pediatric age groups and emphasize the urgent initiation of effective antifungal therapy, surgical debridement, and management of underlying risk factors. l-AmB is strongly recommended for CNS involvement. d-AmB serves as an alternative in neonates when lipid formulations are unavailable.

Recommended pediatric dosin:g^3^ Induction Therapy with l-AmB at dose between 5 and less of 10 mg/kg/day is strongly recommended. Doses approaching 10 mg/kg/day may be warranted for CNS involvement. Combination therapy, such as AmB with echinocandins or triazoles (ISA or posaconazole), may be considered for rescue treatment.^96^, ^97^, ^98^ Sequential Therapy: l-AmB administered 1–3 times weekly or triazoles tailored to age-specific recommendations. While ISA is not yet approved for pediatric use in Brazil, it is utilized internationally. These cases should ideally be managed by pediatric infectious disease specialists.

**2) Surgical debridement with adequate safety margins:** Surgical interventions, often involving multiple specialties such as otorhinolaryngology, ophthalmology, head and neck surgery, thoracic surgery, neurosurgery, and gastrointestinal surgery, are critical for the management of mucormycosis. Early surgical intervention has been associated with improved cure and survival rates in several studies.^3^^,^^7^^,^^10^^,^^21^^,^^25^^,^^34^^,^^40^^,^^92^^,^^99^, ^100^, ^101^, ^102^, ^103^, ^104^, ^105^, ^106^, ^107^, ^108^, ^109^, ^110^, ^111^, ^112^ Debridement with adequate safety margins is strongly recommended whenever possible.^3^ The staging of the mycosis and the patient's preoperative clinical condition must be thoroughly assessed. Surgical approaches vary based on the clinical form and anatomical location of the infection, including debridement of skin and soft tissues, ROC debridement with orbital exenteration, pulmonary resections, bone debridement, and visceral resections of the liver, spleen, peritoneal structures, or transplanted organs. Close clinical and radiological monitoring is essential, as successive debridements may be required if new areas of necrosis are identified.

**3) Prompt control of predisposing factors:** Immediate management of underlying conditions, such as hyperglycemia, metabolic acidosis, and immunosuppression, is essential to optimize patient outcomes and halt disease progression.^3^^,^^72^

## Perspectives

Improving patient outcomes in mucormycosis will require a coordinated and multifaceted approach. A central priority is the strengthening of diagnostic capacity across Brazil, particularly in public hospitals. This includes expanding the use of molecular assays in blood, bronchoalveolar lavage fluid, and biopsy specimens, as well as improving species-level identification through molecular biology techniques and MALDI-TOF platforms. Early and accurate diagnosis remains the cornerstone of reducing mortality, however, most institutions still depend on delayed histopathological or culture-based confirmation.

In addition, the Brazilian Ministry of Health has consolidated a formal program to regulate antifungal access within the public health system. According to Informative Note No 9/2023-CGTM/DATHI/SVSA/MS, a structured flow now governs the request and distribution of strategic antifungals, including l-AmB and ISA. The extended release posaconazole tablet is in the process of being submitted to Conitec for incorporation into the SUS, thus expanding therapeutic options. Requests must meet strict criteria, including laboratory-confirmed diagnosis, completion of standardized request forms. All submissions are reviewed by a technical team from the Coordination of Tuberculosis, Endemic Mycoses, and Nontuberculous Mycobacteria (CGTM/MS), in alignment with national and international clinical guidelines. Beyond drug supply, recent governmental initiatives have supported the acquisition of inputs, funding of research, development of distance-learning courses, and implementation of the MICOSIS platform, a digital system designed to receive antifungal requests and facilitate case notification. Since 2024, a Technical Advisory Committee has been established, and pilot surveillance systems have been launched in the states of Paraná, Mato Grosso do Sul, and São Paulo. In 2025, national expansion is planned to strengthen epidemiological surveillance and standardize fungal infection management throughout the country.

Pediatric mucormycosis remains a neglected area. Current treatment recommendations are predominantly extrapolated from adult data, underscoring the urgent need for pediatric-specific clinical trials and national registry-based studies to support age-appropriate management protocols.

From a pharmacological standpoint, the development of novel antifungal agents represents an important frontier in the management of mucormycosis. Emerging compounds such as fosmanogepix, which has demonstrated in vitro and in vivo activity against Mucorales, are currently undergoing clinical trials and have shown promising results.^113^ Although not yet approved or commercially available in Brazil, their future incorporation into therapeutic protocols may broaden treatment options, especially in cases refractory to existing agents.

Ultimately, sustained collaboration among healthcare professionals, researchers, public health agencies, and policymakers is needed. By investing in diagnostic infrastructure, antifungal access, pediatric research, surveillance, and professional training, and building on existing international evidence, Brazil can take critical steps toward reducing the burden of mucormycosis.

## Conclusion

Mucormycosis remains a challenge in infectious disease management due to its high mortality, diagnostic complexity, and the need for aggressive treatment strategies. This review highlights the critical importance of early diagnosis, multidisciplinary care, and the integration of advanced antifungal therapies, including l-AmB and ISA, as endorsed by Brazil's public health initiatives. Furthermore, the establishment of a specialized advisory committed underlines the country's commitment to addressing the burden of mucormycosis through evidence-based approaches. Future efforts should prioritize the development of rapid diagnostic tools, improved therapeutic options, and systematic surveillance to reduce the impact of this devastating opportunistic infection.

All authors have read and agreed to the published version of the manuscript.

## Consent for publication

All authors have read and agreed to the published version of the manuscript.

## Institutional review board statement

The study was conducted in accordance with the Declaration of Helsinki.

## Funding

The authors did not receive specific funding for this study.

## Availability of data and material

Not applicable.

## Conflicts of interest

M.M.C.M has received support for attending educational meetings from Knight and Mundipharma. The remaining authors declare no conflict of interest.
